# Body-weight variability and risk of cardiovascular outcomes in patients with type 1 diabetes: a retrospective observational analysis of data from the DCCT/EDIC population

**DOI:** 10.1186/s12933-022-01689-0

**Published:** 2022-11-17

**Authors:** Iulia Petria, Samuel Albuquerque, Gaël Varoquaux, Jill-Jênn Vie, Nicolas Venteclef, Kamel Mohammedi, Ronan Roussel, Marion Camoin, Gianluca Perseghin, Gilberto Velho, Louis Potier

**Affiliations:** 1 School of Medicine and Surgery, University of Milano-Bicocca, Monza, Italy; 2 Institut Necker-Enfants Malades, Université Paris Cité, INSERM UMR-S1151, CNRS UMR-S8253, Paris, France; 3 SODA, INRIA, Saclay, France; 4 Université Paris Cité, UFR de Médecine, Paris, France; 5 Bordeaux University and Hospital, INSERM U1034, Bordeaux, France; 6 Department of Diabetology, Endocrinology and Nutrition, Assistance Publique - Hôpitaux de Paris, Bichat Hospital, Paris, France; 7 Department of Medicine and Rehabilitation, Policlinico di Monza, Monza, Italy

**Keywords:** Cardiovascular risk, Mortality, Type 1 diabetes, Weight variation

## Abstract

**Background:**

Cardiovascular risk and body-weight management are both emerging challenges of type 1 diabetes care. We evaluated the association between intraindividual variability of body-weight and risk of cardiovascular events in people with type 1 diabetes.

**Methods:**

We analyzed 1,398 participants from the DCCT/EDIC studies. Five indices of intraindividual variability of body-weight were calculated for each participant taking into account body-weight measures obtained during the DCCT follow-up (average 6 ± 2 years). The Average Successive Variability (ASV) index, the main variable of interest, was defined as the average absolute difference between successive body-weight measures. The primary outcome was a composite of major adverse cardiovascular events (MACE: nonfatal myocardial infarction or stroke, or cardiovascular death) occurring during the subsequent EDIC follow-up (20 ± 3 years). All-cause death was a secondary outcome. Risk of outcomes were assessed by Cox proportional hazards regression analyses, adjusted for traditional cardiovascular risks factors, including BMI.

**Results:**

The cumulative incidence of MACE and all-cause death during follow-up were 5.6% (n = 79) and 6.8% (n = 95), respectively. The adjusted Hazard Ratio (HR) for MACE by every increase of 1 standard deviation (SD) of ASV was 1.34 (95% CI, 1.06–1.66), p = 0.01. For all-cause death, the adjusted HR for 1 SD increase of ASV was 1.25 (1.03–1.50), p = 0.03. Similar results were observed when considering the other indices of intraindividual variability of body-weight.

**Conclusions:**

High body-weight variability (body-weight cycling) is associated with increased risk of MACE and all-cause death in people with type 1 diabetes, independently of the BMI and traditional cardiovascular risk factors.

**Supplementary Information:**

The online version contains supplementary material available at 10.1186/s12933-022-01689-0.

## Background

The major advances in diabetes management and care over the last decades [1] have led to a reduction in mortality in people with type 1 diabetes [2, 3]. However, premature mortality in type 1 diabetes still exceeds by 2–fourfold that of the general population [4]. Acute glycemic-related complications such as ketoacidosis and hypoglycemia, and long-term microvascular complications, notably end-stage kidney disease, have historically accounted for most deaths in type 1 diabetes [5]. However, data from diverse geographic regions, including Northern Europe [6, 7] and the USA [5, 8], suggest that macrovascular complications are a significant cause of morbidity and mortality in these patients. Thus, assessing cardiovascular risk factors in people with type 1 diabetes is a crucial challenge to optimize disease management and prevent these adverse outcomes [9].

The Diabetes Control and Complications Trial (DCCT) [10] and the Epidemiology of Diabetes Interventions and Complications (EDIC) [11] are milestone studies on both microvascular and macrovascular complications in type 1 diabetes. The implication of a great number of participants across multiple centers and the extension of follow-up make the DCCT/EDIC database an invaluable resource of type 1 diabetes data [12].

Excessive weight gain was associated with measures of subclinical atherosclerosis and predictors of cardiovascular events and with higher rates of cardiovascular events in the DCCT/EDIC studies [13, 14]. Fluctuation of body-weight, also known as weight cycling, i.e., repeatedly losing and regaining weight [15], was shown to be an independent predictor of cardiovascular events and mortality risk in the general population [16, 17], as well as in people with coronary artery disease [18], or type 2 diabetes [19, 20]. Weight cycling is a potential non-traditional cardiovascular risk factor in patients with type 1 diabetes. However, to our knowledge, there are no studies evaluating the relationship between weight variability and cardiovascular risk in this population. In the present investigation we assessed associations between body-weight fluctuation and cardiovascular events in type 1 diabetes through a secondary analysis of data from the DCCT/EDIC population. We also evaluated the impact of the DCCT study treatment allocation in these associations.

## Methods

### Study population

We conducted a retrospective observational analysis of data from the DCCT/EDIC studies (NCT00360815 and NCT00360893). The provision and use of the publicly available DCCT and EDIC data sets were requested and obtained from the National Institute of Diabetes and Digestive and Kidney Diseases. In brief, the DCCT recruited 1,441 volunteers with type 1 diabetes, aged between 13 and 39, and followed them for an average of 6 year, from 1983 to 1993 [10]. In that randomized, controlled study participants were assigned to receive either intensive therapy, aimed at achieving glycemic control as close to the nondiabetic range as safely possible, or conventional therapy. The goals of conventional therapy included the absence of symptoms attributable to glycosuria or hyperglycemia, the absence of ketonuria, the maintenance of normal growth, development, and ideal body weight, and freedom from severe or frequent hypoglycemia [10]. The primary DCCT outcome was the incidence of retinopathy, but renal, cardiovascular, neurologic and neuropsychological outcomes were also studied. The DCCT demonstrated the powerful impact of intensive glycemic control on the early manifestations of microvascular complications. EDIC was a post-trial observational follow-up study of the DCCT cohort to aiming to assess the effects of intensive therapy on the more advanced stages of complications and cardiovascular disease [11]. Starting in 1994, annual or biennial assessments of extensive clinical and biological data were collected over two decades using DCCT methods, standardized protocols and central laboratories. We analyzed 1,398 participants of the DCCT/EDIC studies, including 1212 adults and 186 teenagers younger than 18 years. Participants with no available data during the EDIC follow-up (n = 34) or for whom less than four measurements of body-weight were available during the DCCT follow-up (n = 9) were excluded from the present investigation. Participants with major adverse cardiovascular events (MACE, see definition below, n = 4) during the DCCT follow-up were excluded from the analyses of incident MACE during the EDIC follow-up. The flowchart of participants is shown in the Additional file 1: Fig. S1.

### Clinical parameters and outcomes

The information collected included sex, age, body weight, BMI, blood pressure, HbA1c, blood lipids, serum creatinine, urinary albumin excretion (UAE), and use of antihypertensive and lipid-lowering drugs. Arterial hypertension was defined as systolic blood pressure > 140 mmHg or diastolic blood pressure > 90 mmHG or use of an antihypertensive drug. Hyperlipidemia was defined as LDL-cholesterol ≥ 3.37 mmol/L (130 mg/dl) or use of a lipid-lowering drug. The estimated glomerular filtration rate (eGFR) was computed with CKD-EPI study equation for serum creatinine [21]. Data on tobacco smoking was available as a categorical variable: non-smoker, occasional smoker, or regular smoker. The last two categories were considered as indicative of smoking status.

Five indices of intraindividual variability of body weight [18] were calculated for each participant taking into account yearly body-weight measures obtained from the second year post-inclusion to the end of the DCCT follow-up. Body-weight measures from the first year following study treatment allocation were discarded from the computations to avoid bias of rapid changes in glycemic control on body-weight [22]. The main variability index of interest was the Average Successive Variability (ASV), defined as the average absolute difference between successive body-weight values:

$$ASV= \frac{\left|{weight}_{1}- {weight}_{0}\right| + \left|{weight}_{2}- {weight}_{1}\right| + \cdots + \left|{weight}_{n}- {weight}_{n-1}\right|}{n-1},$$

where ${weight}_{0, 1,\cdots ,n}$ indicates body-weight measurements at visits $\mathrm{0,1},\dots , n$.

The other indices of body-weight variability were:

- The standard deviation of body-weight measures (SD_bw)
- The coefficient of variation (CVAR), defined as $\frac{standard\,deviation\,of\,body\,weight\,measures}{mean\,of\,body\,weight\,measures}$
- The relative ASV (REL_ASV), defined as $\frac{ASV}{initial\,body\,weight}$.
- The variability independent of the mean (VIM), defined as $\frac{standard\,deviation\,of\,body\,weight\,measures}{{mean\,of\,body\,weight\,mesures}^{beta}}$, where beta is the regression coefficient of the correlation of the natural logarithm of the standard deviation (y axis) on the natural logarithm of the mean (x axis).

The primary outcome of the study was a composite of MACE, defined as the first occurrence of nonfatal myocardial infarction or stroke, or cardiovascular death during the EDIC follow-up [23]. The secondary outcome was death from all causes during the EDIC follow-up.

### Statistical analyses

Categorical variables were expressed as number of participants with corresponding percentage. Continuous variables were expressed as mean ± SD or as median and interquartile range (IQR) for those with skewed distribution. Characteristics of participants at baseline were compared between groups by Pearson's chi-squared test, Fisher's exact test, Student's t test, Kruskal–Wallis test or ANOVA. Cox proportional hazards regression analysis models were fitted to estimate associations of indices of intraindividual variability of body-weight with the outcomes. Hazard Ratios (HR) with associated 95% confidence interval (CI) were computed in these analyses for 1 SD of the indices expressed as a Z-score. Cox analyses were adjusted for sex, baseline DCCT body-weight, study treatment allocation during the DCCT, baseline EDIC characteristics, including age, sex, duration of diabetes, eGFR, presence of hyperlipidemia and arterial hypertension, tobacco smoking, and for time-dependent covariates expressed as the average value of yearly measures during DCCT + EDIC follow-up until the first outcome occurrence, comprising BMI, systolic blood pressure, circulating levels of HbA1c, LDL-cholesterol and triglycerides, and UAE. To standardize the results of the Cox analyses, continuous covariates were entered in the regression model as a Z-score, and those with skewed distribution (duration of diabetes, triglycerides and UAE) as a Z-score of log-transformed data. We checked that the Cox regression model validates the proportional hazards assumption (Schoenfeld residuals) and that no major collinearity issues were observed (variance inflation factors of all covariates < 2.25). To assess the impact of DCCT study treatment on the association of indices of intraindividual variability of body weight with the outcomes, Cox analyses were also performed in subsets of participants stratified by the DCCT allocation (conventional or intensive treatment). Sensitivity analyses were also performed in the subset of adult participants, aged 18 years or older at DCCT baseline (n = 1212). For an additional set of sensitivity analyses, the indices of intraindividual variability of body weight were recalculated with the inclusion of body-weight measures from the first year following study treatment allocation. Statistics were performed with Python (version 3.8.8, https://www.python.org) using the Lifelines library (version 0.27.0, https://lifelines.readthedocs.io) [24] and with JMP (version 16, www.jmp.com). P < 0.05 (two sided) was considered significant.

## Results

### MACE during EDIC follow-up by indices of intraindividual variability of body-weight during the DCCT

Indices of intraindividual variability of body-weight were computed from 6.3 ± 1.6 intraindividual body-weight measures during the DCCT. Duration of the DCCT follow-up was 6 ± 2 years. The last DCCT follow-up visit was considered as the EDIC baseline, and the average duration of the EDIC follow-up was 20 ± 3 years. Myocardial infarction, stroke and cardiovascular death occurred during the EDIC follow-up in 49 (3.5%), 19 (1.4%) and 18 (1.3%) of the participants selected for the present study, respectively, with 7 participants presenting more than one event. The cumulative incidence of MACE during follow-up was 5.7% (n = 79), and its incidence rate per 1000 person-years was 2.9. Characteristics of participants at EDIC baseline by the incidence of MACE during follow-up are summarized in the Additional file 1: Table S1. Baseline characteristics by tertiles of the ASV index distribution are summarized in Table 1. Briefly, participants in the higher tertile of ASV (T3, higher body-weight variability) as compared to those in the lower tertiles were more likely to be women, were younger, had a higher BMI and higher circulating levels of total cholesterol, LDL-cholesterol and triglycerides, a higher eGFR, and were more likely to have been allocated to the DCCT study intensive treatment group. All indices of variability of body weight were higher in the intensive treatment group than in the standard treatment group (Table 2).

**Table 1:** Characteristics of participants at EDIC baseline by tertiles of ASV distribution

	All	ASV Tertiles			P
		T1	T2	T3	
N	1,398	465	468	465	
ASV, kg*	2.33 [1.69]	1.43 [0.52]	2.33 [0.48]	3.90 [1.58]	< 0.0001
DCCT group: intensive treatment, n (%)	693 (50)	187 (40)	225 (48)	281 (60)	< 0.0001
Duration of DCCT follow-up, years	6.2 ± 1.8	6.0 ± 1.7	6.4 ± 1.8	6.1 ± 1.8	0.0002
Duration of EDIC follow-up, years	20.0 ± 3.3	20.1 ± 3.0	20.0 ± 3.3	19.9 ± 3.5	0.50
Sex: male, n (%)	733 (52)	260 (56)	265 (57)	208 (45)	0.0002
Age, years	33 ± 7	34 ± 7	34 ± 7	32 ± 7	< 0.0001
BMI, kg/m^2^	25.8 ± 3.8	24.2 ± 2.7	25.8 ± 3.2	27.4 ± 4.7	< 0.0001
Duration of diabetes, years	12 ± 5	12 ± 5	13 ± 5	12 ± 5	0.0005
HbA1c, %	8.3 ± 1.6	8.3 ± 1.6	8.3 ± 1.6	8.2 ± 1.7	0.24
HbA1c, mmol/mol	67 ± 18	68 ± 17	67 ± 17	66 ± 19	0.24
Systolic BP, mmHg	117 ± 12	117 ± 12	117 ± 12	116 ± 11	0.28
Diastolic BP, mmHg	75 ± 9	74 ± 9	75 ± 9	74 ± 9	0.25
Arterial hypertension, n (%)	57 (4.1)	11 (2.4)	26 (5.6)	20 (4.3)	0.05
Total cholesterol, mmol/L	4.72 ± 0.89	4.65 ± 0.85	4.68 ± 0.80	4.82 ± 0.96	0.007
LDL-cholesterol, mmol/L	2.94 ± 0.77	2.88 ± 0.74	2.92 ± 0.76	3.02 ± 0.80	0.02
HDL-cholesterol, mmol/L	1.33 ± 0.34	1.35 ± 0.35	1.32 ± 0.34	1.32 ± 0.32	0.20
Triglycerides, mmol/L*	0.83 [0.49]	0.75 [0.48]	0.83 [0.52]	0.88 [0.53]	< 0.0001
Hyperlipidemia, n (%)	387 (28)	106 (23)	139 (30)	142 (31)	0.01
eGFR, ml/min/1.73m^2^	117 ± 13	116 ± 12	116 ± 12	119 ± 14	0.008
UAE, mg/24 h*	10 [12]	9 [9]	10 [13]	9 [13]	0.02
UAE > 30 mg/24 h, n (%)	149 (11)	41 (9)	66 (14)	42 (9)	0.01
Tobacco smoking, n (%)	278 (20)	83 (18)	88 (19)	107 (23)	0.10
Family history of MI, n (%)	681 (49)	220 (47)	230 (49)	231 (50)	0.74
Family history of T2D, n (%)	126 (9)	38 (8)	41 (9)	47 (10)	0.57

Data from EDIC baseline, unless stated otherwise. Continuous data expressed as mean ± SD or as median [IQR]*. Categorical data expressed as number (%). Statistics are ANOVA, *Kruskal–Wallis test or Pearson's chi-squared test. ASV: Average Successive Variability index of body-weight variability during DCCT follow-up. Arterial hypertension: Systolic BP > 140 mmHg or Diastolic BP > 90 mmHG or use of antihypertensive drug. Hyperlipidemia: LDL-cholesterol 3.37 ≥ mmol/L (130 mg/dl) or use of lipid-lowering drug. *eGFR* estimated glomerular filtration rate. *UAE* Urinary albumin excretion. *MI* myocardial infarction. *T2D* type 2 diabetes

**Table 2:** Indices of intraindividual variability of body-weight by study treatment allocation during the DCCT

	ALL	Conventional treatment	Intensive treatment	p
ASV	2.68 ± 1.52	2.47 ± 1.43	2.90 ± 1.58	< 0.0001
SD_bw	3.24 ± 2.01	2.83 ± 1.69	3.66 ± 2.21	< 0.0001
CVAR × 100	4.34 ± 2.54	3.96 ± 2.45	4.72 ± 2.59	< 0.0001
REL_ASV × 100	3.80 ± 2.20	3.58 ± 2.21	4.03 ± 2.17	< 0.0001
VIM × 100	2.70 ± 1.59	2.48 ± 1.54	2.93 ± 1.60	< 0.0001

Data are mean ± SD. Statistics are Student's t test. See methods for the definitions of the indices of intraindividual variability of body-weight

In Cox analyses adjusted for confounding covariates, the five indices of variability of body-weight were significantly and positively associated with the incidence of MACE (Table 3 and Additional file 1: Fig. S2). Upon stratification by DCCT study treatment allocation, the associations were significant only in the conventional treatment group. In sensitivity analysis of the adult population only, the five indices of intraindividual variability of body-weight remained significantly associated with the outcome (Additional file 1: Table S2). Associations with MACE remained significant in the whole population and in the conventional treatment group in additional sensitivity analyses with the indices of intraindividual variability of body weight recalculated with the inclusion of body-weight measures from the first year following study treatment allocation (Additional file 1: Table S3 and Fig. S2).

**Table 3:** Risk of outcomes during EDIC follow-up by indices of intraindividual variability of body-weight during the DCCT

	All participants		Conventional treatment		Intensive treatment	
	HR (95% C.I.)	p	HR (95% C.I.)	p	HR (95% C.I.)	p
MACE						
ASV	1.34 (1.06–1.66)	0.01	1.50 (1.07–2.05)	0.02	1.15 (0.79–1.62)	0.45
SD_bw	1.55 (1.20–1.96)	0.0009	1.79 (1.22–2.55)	0.004	1.40 (0.97–1.98)	0.08
CVAR	1.50 (1.18–1.88)	0.001	1.65 (1.18–2.26)	0.005	1.40 (0.94–2.03)	0.09
REL_ASV	1.32 (1.03–1.66)	0.03	1.45 (1.03–1.97)	0.03	1.11 (0.72–1.63)	0.63
VIM	1.50 (1.17–1.88)	0.002	1.64 (1.17–2.23)	0.005	1.40 (0.94–2.04)	0.10
All-cause death						
ASV	1.25 (1.03–1.50)	0.03	1.50 (1.17–1.89)	0.002	1.05 (0.73–1.46)	0.78
SD_bw	1.42 (1.14–1.76)	0.003	1.69 (1.27–2.18)	0.0005	1.18 (0.82–1.66)	0.37
CVAR	1.39 (1.11–1.70)	0.004	1.59 (1.22–2.02)	0.0009	1.13 (0.78–1.60)	0.50
REL_ASV	1.32 (1.08–1.60)	0.008	1.59 (1.23– 2.01)	0.0006	1.10 (0.76–1.54)	0.60
VIM	1.38 (1.11–1.69)	0.005	1.59 (1.22–2.01)	0.001	1.13 (0.78–1.59)	0.51

Hazard Ratios computed by Cox proportional hazards survival regression analyses for 1 SD of the body-weight variability indices expressed as Z-scores. Regression models adjusted for sex, baseline DCCT body-weight, study treatment allocation during the DCCT, baseline EDIC characteristics, including age, sex, duration of diabetes, eGFR, presence of hyperlipidemia and arterial hypertension, tobacco smoking, and for time-dependent covariates expressed as the average value during DCCT + EDIC follow-up until the first outcome occurrence, comprising BMI, systolic blood pressure, circulating levels of HbA1c, LDL-cholesterol and triglycerides, and UAE. See methods for the definitions of the indices of intraindividual variability of body-weight

### All-cause death during EDIC follow-up by indices of intraindividual variability of body-weight during the DCCT

The cumulative incidence of all-cause death during follow-up was 6.8% (n = 95), and its incidence rate was 3.4 per 1000 person-years. In adjusted Cox analyses, the five indices of body-weight variability expressed as Z-scores were significantly and positively associated with all-cause death during follow-up (Table 3 and Additional file 1: Fig. S3). Upon stratification by DCCT study treatment allocation, the associations were significant only in the conventional treatment group. In sensitivity analysis of the adult population only, none of the indices remained significantly associated with the outcome in the whole population (Additional file 1: Table S2), but the associations remained significant in the conventional treatment group. Associations of the five indices of intraindividual variability of body weight with the outcome were also observed for the indices recalculated with the inclusion of body-weight measures from the first year following study treatment allocation (Additional file 1: Table S3 and Fig. S3). Upon stratification by DCCT study treatment allocation, the associations were significant only in the conventional treatment group.

## Discussion

The present retrospective analysis of the DCCT/EDIC data documents associations between a high intra-individual variability in body weight during the DCCT study and increased risk of MACE and all-cause mortality during the subsequent EDIC follow-up. The associations were independent of BMI and of relevant cardiovascular risk factors. DCCT/EDIC investigators reported associations of weight gain during the DCCT with increased total cardiovascular events during the EDIC follow-up [14]. However, to the best of our knowledge, this is the first report of associations between increased body weight cycling and a high risk of cardiovascular events and mortality in people with type 1 diabetes.

As in any epidemiological study, observed associations may be confounded by several factors. A history of cardiovascular disease at inclusion in prospective studies can be a major risk factor for cardiovascular events during follow-up, often masking the effects of intermediate risk factors. By study design, DCCT participants were free of cardiovascular complications at inclusion, and in the present investigation, we have excluded incident cases of MACE during the DCCT from the MACE analyses during EDIC follow-up.

Intensive insulin therapy during the DCCT was associated with weight gain, mainly in the first year after inclusion [22, 25]. In the main analyses of this investigation, body-weight measures taken during the first year of DCCT follow-up were not used in the computations of the indices of variability of body weight, in order to avoid the impact of early change in glycemic control on body weight. In addition, the DCCT study treatment group was systematically included as a covariate in the analyses. It is noteworthy that associations with MACE presented a lesser degree of statistical significance in sensitivity analyses taking into account body-weight measures of the first year of DCCT follow-up in the computations of the indices. However, the DCCT study treatment had a major impact on the associations of the indices of variability of body weight with the outcomes, as the associations were significant in the conventional treatment group, but not in the intensive treatment group. This observation is paradoxical because the indices of variability of body weight were significantly higher in the intensive treatment group than in the standard treatment group. We don't have a clear explanation for this paradoxical observation. It does not seem to be related to a lack of statistical power as not even a trend towards association was observed in the intensive treatment group. It is possible that the conditions or mechanisms associated with or leading to body-weight variability were different in the two groups of participants, affecting differently the risk of outcomes. It is also plausible to speculate that the deleterious effect of body-weight cycling on the risk of outcomes in the intensive treatment group might have been compensated by favorable effects of intensive insulin treatment and better glycemic control on the outcomes [26].

Type 1 diabetes was traditionally considered as a disease of lean individuals, but overweight and obesity, increasingly prevalent in the general population, are also becoming more prevalent in people with type 1 diabetes [27]. The causes of weight cycling in people with type 1 diabetes are probably complex. Environmental, pharmacological and heritable factors, as well as behavioral and lifestyle factors are known to affect weight gain and weight loss in the general population. [27]. In addition, the use of insulin in type 1 diabetes might be an important driver of body-weight cycling. Less than optimal insulin therapy is associated with weight loss, while an improved glycemic control is associated with weight gain [10] as a direct consequence of the anabolic effects of insulin. Moreover, the risk of hypoglycemia associated with insulin treatment might lead to defensive snacking and less physical exercise [27]. We have previously reported that the frequent non-severe episodes of hypoglycemia observed during intensive glucose control in DCCT participants were associated with subsequent weight gain [28].

The pathophysiological mechanisms behind the association of body weight cycling and cardiovascular risk are largely unknown. Several non-exclusive mechanisms, including low-grade inflammation, insulin resistance and oxidative stress, have been evoked as possible links. Body-weight fluctuations may result in relative hypoxia and chronic low-grade inflammation in the adipose tissue, leading to accumulation and activation of macrophage, and the release of pro-inflammatory cytokines by macrophages and hypoxic adipocyte [29, 30]. An animal model study showed weight cycling to be associated with decreased glucose tolerance and insulin sensitivity. The phenotype was mediated in the adipose tissue by decreased expression and phosphorylation of the insulin signaling molecules PI3K and PKB, decreased expression of GLUT4, downregulation of the anti-inflammatory adipokine CTRP3 expression, and upregulation of the pro-inflammatory cytokines IL-6 and TNF-alpha [31]. However, because of its observational nature, our study cannot preclude or confirm any of these mechanisms.

The main strength of the present investigation was the analysis of a group of well-characterized patients, with a relatively long duration of follow-up within the structured clinical protocol of the DCCT/EDIC studies. There are limitations of the study to acknowledge. Firstly, its design did not allow any conclusion on the possible causal relationship between body weight cycling and the outcomes. Secondly, due to the relatively small number of MACE events, we had to use a composite outcome to ensure a minimal number of cardiovascular events during follow-up. Acute myocardial infarction, stroke and cardiovascular death may be heterogeneous regarding weight-cycling implication, and the study did not have sufficient power to assess such heterogeneity. Thirdly, there was no information on the underlying cause of body-weight variability. In a meta-analysis, intentional weight loss by lifestyle interventions was associated with a reduction of cardiovascular events, whereas unintentional weight loss was associated with increased event rates [32].

## Conclusions

In conclusion, we observed associations between high body-weight variability and increased risk of MACE and all-cause mortality in patients with type 1 diabetes from the DCCT/EDIC studies. The associations were independent of the BMI and of series of traditional cardiovascular risk factors, and they confirm similar observations in the general population. However, the associations were significant in the conventional treatment group, but not in the intensive treatment group. Cardiovascular risk and body-weight management are both emerging challenges of type 1 diabetes care. Our findings suggest that weight fluctuations may lead to negative health outcomes in the setting of less than optimal glucose control. Further investigations are needed to confirm and clarify this interaction. From a clinical point of view, strategies to reduce body weight in people with type 1 diabetes should encompass the promotion of durable maintenance of weight loss, as the stability of weight could positively influence health outcomes.

## Supplementary Information

**Additional file 1:** **Table S1.** Characteristics of participants at EDIC baseline by the incidence of MACE during EDIC follow-up. **Table S2. **Risk of outcomes in adult participants during EDIC follow-up by indices of intraindividual variability of body-weight during the DCCT. **Table S3.** Risk of outcomes during EDIC follow-up by indices of intraindividual variability of body-weight computed with body-weight measures from baseline to the end of DCCT follow-up. **Figure S1.** Flowchart of participants. **Figure S2.** Forest plots for the risk of MACE during EDIC follow-up by indices of intraindividual variability of body-weight during the DCCT. **Figure S3.** Forest plots for the risk of all-cause death during EDIC follow-up by indices of intraindividual variability of body-weight during the DCCT.

## Data Availability

Data from DCCT/EDIC are publicly available on the NIDDK Central Repository.

## Abbreviations

ASV: Average Successive Variability
CVAR: Coefficient of variation
DCCT: Diabetes Control and Complications Trial
EDIC: Epidemiology of Diabetes Interventions and Complications
eGFR: Estimated glomerular filtration rate
MACE: Major adverse cardiovascular events
REL_ASV: Relative ASV
SD_bw: Standard deviation of body-weight measures
UAE: Urinary albumin excretion
VIM: Variability Independent of the Mean
