# Supplementary material for: Body-weight variability and risk of cardiovascular outcomes in patients with type 1 diabetes: a retrospective observational analysis of data from the DCCT/EDIC population
Source: Cardiovasc Diabetol. 2022 Nov 17;21:247. doi: 10.1186/s12933-022-01689-0 (PMC9670666; doi:10.1186/s12933-022-01689-0)
Supplement: Supplementary file 1 — Additional file 1: Table S1. Characteristics of participants at EDIC baseline by the incidence of MACE during EDIC follow-up. Table S2. Risk of outcomes in adult participants during EDIC follow-up by indices of intraindividual variability of body-weight during the DCCT. Table S3. Risk of outcomes during EDIC follow-up by indices of intraindividual variability of body-weight computed with body-weight measures from baseline to the end of DCCT follow-up. Figure S1. Flowchart of participants. Figure S2. Forest plots for the risk of MACE during EDIC follow-up by indices of intraindividual variability of body-weight during the DCCT. Figure S3. Forest plots for the risk of all-cause death during EDIC follow-up by indices of intraindividual variability of body-weight during the DCCT. [file 12933_2022_1689_MOESM1_ESM.docx]

**Body-Weight Variability and Risk of Cardiovascular Outcomes in Patients with Type 1 Diabetes: A Retrospective Observational Analysis of Data from the DCCT/EDIC Population**

**Supplemental Material**

Page 2. Additional file 1: Table S1. Characteristics of participants at EDIC baseline by the incidence of MACE during EDIC follow-up

Page 3 Additional file 1: Table S2. Risk of outcomes in adult participants during EDIC follow-up by indices of intraindividual variability of body-weight during the DCCT

Page 4 Additional file 1: Table S3. Risk of outcomes during EDIC follow-up by indices of intraindividual variability of body-weight computed with body-weight measures from baseline to the end of DCCT follow-up

Page 5 Additional file 1: Fig. S1. Flowchart of participants

Page 6 Additional file 1: Fig. S2. Forest plots for the risk of MACE during EDIC follow-up by indices of intraindividual variability of body-weight during the DCCT.

Page 7 Additional file 1: Fig. S3. Forest plots for the risk of all-cause death during EDIC follow-up by indices of intraindividual variability of body-weight during the DCCT.

**Additional file 1: Table S1. Characteristics of participants at EDIC baseline by the incidence of MACE during EDIC follow-up**

|  | **MACE during follow-up** | | **p** |
| --- | --- | --- | --- |
|  | **No** | **Yes** |  |
| N | 1315 | 79 |  |
| Sex: male, n (%) | 686 (52) | 44 (56) | 0.56 |
| Age, years | 33 ± 7 | 38 ± 5 | <0.0001 |
| BMI, kg/m^2^ | 25.8 ± 3.8 | 26.1 ± 4.3 | 0.45 |
| Duration of diabetes, years | 12 ± 5 | 14 ± 5 | 0.006 |
| HbA1c, % | 8.2 ± 1.6 | 8.9 ± 1.9 | 0.001 |
| HbA1c, mmol/mol | 67 ± 18 | 73 ± 20 | 0.001 |
| Systolic BP, mmHg | 116 ± 12 | 121 ± 11 | 0.001 |
| Diastolic BP, mmHg | 74 ± 9 | 77 ± 8 | 0.009 |
| Arterial hypertension, n (%) | 51 (3.9) | 5 (6.3) | 0.24 |
| Total cholesterol, mmol/L | 4.69 ± 0.88 | 5.06 ± 0.88 | 0.0004 |
| LDL-cholesterol, mmol/L | 2.92 ± 0.76 | 3.23 ± 0.76 | 0.0004 |
| HDL-cholesterol, mmol/L | 1.33 ± 0.34 | 1.30 ± 0.33 | 0.39 |
| Triglycerides, mmol/L* | 0.83 [0.49] | 1.02 [0.68] | <0.0001 |
| Hyperlipidemia, n (%) | 349 (27) | 36 (46) | 0.0006 |
| eGFR, ml/min/1.73m^2^ | 117 ± 13 | 114 ± 13 | 0.02 |
| UAE, mg/24h* | 10 [10] | 10 [22] | 0.37 |
| UAE > 30 mg/24h, n (%) | 131 (10) | 17 (22) | 0.004 |
| Tobacco smoking, n (%) | 249 (19) | 27 (34) | 0.002 |
| Family history of MI, n (%) | 639 (49) | 42 (53) | 0.49 |
| Family history of T2D, n (%) | 119 (9) | 7 (9) | 0.99 |

Data from EDIC baseline. Continuous data expressed as mean ± SD or as median [IQR]*. Categorical data expressed as number (%). Statistics are Student's t test, *Kruskal-Wallis test, or Fisher's exact test. Arterial hypertension: Systolic BP >140 mmHg or Diastolic BP >90 mmHG or use of antihypertensive drug. Hyperlipidemia: LDL-cholesterol ≥3.37 mmol/L (130 mg/dl) or use of lipid-lowering drug. eGFR: estimated glomerular filtration rate. UAE: Urinary albumin excretion. Tobacco smoking: current or ex-smokers. MI: myocardial infarction. T2D: type 2 diabetes.

**Additional file 1: Table S2. Risk of outcomes in adult participants during EDIC follow-up by indices of intraindividual variability of body-weight during the DCCT**

|  |  | **MACE*** | |  | **All-cause Death**** | |
| --- | --- | --- | --- | --- | --- | --- |
|  |  | **Hazard Ratio (95% C.I.)** | **p** |  | **Hazard Ratio (95% C.I.)** | **p** |
| ASV |  | 1.34 (1.06 – 1.66) | 0.02 |  | 1.16 (0.94 – 1.40) | 0.17 |
| SD_bw |  | 1.62 (1.26 – 2.07) | 0.0003 |  | 1.20 (0.92 – 1.55) | 0.18 |
| CVAR |  | 1.56 (1.21 – 1.97) | 0.0007 |  | 1.17 (0.90 – 1.49) | 0.23 |
| REL_ASV |  | 1.30 (1.01 – 1.65) | 0.04 |  | 1.18 (0.94 – 1.46) | 0.16 |
| VIM |  | 1.55 (1.21 – 1.96) | 0.0008 |  | 1.17 (0.89 – 1.49) | 0.24 |

Data from 1208* or 1212** adult participants (age ≥18 years) at DCCT baseline. Hazard Ratios computed by Cox proportional hazards survival regression analysis for 1 SD of the indices of intraindividual variability of body-weight expressed as Z-scores. Regression model adjusted for sex, baseline DCCT body-weight, study treatment allocation during the DCCT, baseline EDIC characteristics, including age, sex, duration of diabetes, eGFR, presence of hyperlipidemia and arterial hypertension, tobacco smoking, and for time-dependent covariates expressed as the average value during DCCT + EDIC follow-up until the first outcome occurrence, comprising BMI, systolic blood pressure, circulating levels of HbA1c, LDL-cholesterol and triglycerides, and UAE. See methods for the definitions of the indices of intraindividual variability of body-weight.

**Additional file 1: Table S3. Risk of outcomes during EDIC follow-up by indices of intraindividual variability of body-weight computed with body-weight measures from baseline* to the end of DCCT follow-up**

|  |  | **All participants** | |  | **Conventional treatment** | |  | **Intensive treatment** | |
| --- | --- | --- | --- | --- | --- | --- | --- | --- | --- |
|  |  | **HR (95% C.I.)** | **p** |  | **HR (95% C.I.)** | **p** |  | **HR (95% C.I.)** | **p** |
| **MACE** |  |  |  |  |  |  |  |  |  |
| ASV |  | 1.36 (1.05 – 1.73) | 0.02 |  | 1.61 (1.11 – 2.28) | 0.01 |  | 1.09 (0.72 – 1.60) | 0.67 |
| SD_bw |  | 1.45 (1.08 – 1.93) | 0.01 |  | 1.74 (1.09 – 2.68) | 0.02 |  | 1.27 (0.81 – 1.92) | 0.29 |
| CVAR |  | 1.46 (1.08 – 1.94) | 0.01 |  | 1.66 (1.09 – 2.44) | 0.02 |  | 1.32 (0.81 – 2.10) | 0.27 |
| REL_ASV |  | 1.32 (1.00 – 1.73) | 0.05 |  | 1.53 (1.04 – 2.17) | 0.03 |  | 1.04 (0.64 – 1.63) | 0.88 |
| VIM |  | 1.46 (1.08 – 1.94) | 0.01 |  | 1.66 (1.09 – 2.46) | 0.02 |  | 1.31 (0.81 – 2.08) | 0.26 |
| **All-cause death** |  |  |  |  |  |  |  |  |  |
| ASV |  | 1.33 (1.08 – 1.61) | 0.009 |  | 1.72 (1.32 – 2.21) | 0.0001 |  | 1.04 (0.72 – 1.47) | 0.82 |
| SD_bw |  | 1.51 (1.18 – 1.91) | 0.001 |  | 1.81 (1.30 – 2.45) | 0.0007 |  | 1.37 (0.93 – 1.98) | 0.11 |
| CVAR |  | 1.52 (1.19 – 1.91) | 0.0009 |  | 1.76 (1.29 –2.33) | 0.0006 |  | 1.36 (0.91 – 1.99) | 0.13 |
| REL_ASV |  | 1.46 (1.17 – 1.80) | 0.001 |  | 1.89 (1.43 – 2.45) | <0.0001 |  | 1.13 (0.77 – 1.61) | 0.50 |
| VIM |  | 1.52 (1.19 – 1.91) | 0.0009 |  | 1.80 (1.29 – 2.34) | 0.0006 |  | 1.36 (0.91 – 1.99) | 0.13 |

Hazard Ratios computed by Cox proportional hazards survival regression analysis for 1 SD of the indices of intraindividual variability of body-weight expressed as Z-scores. *Computations of indices of intraindividual variability of body-weight included measures of body-weight from the first year following study treatment allocation. Regression model adjusted for sex, baseline DCCT body-weight, study treatment allocation during the DCCT, baseline EDIC characteristics, including age, sex, duration of diabetes, eGFR, presence of hyperlipidemia and arterial hypertension, tobacco smoking, and for time-dependent covariates expressed as the average value during DCCT + EDIC follow-up until the first outcome occurrence, comprising BMI, systolic blood pressure, circulating levels of HbA1c, LDL-cholesterol and triglycerides, and UAE. See methods for the definitions of the intraindividual variability of body-weight.

**Additional file 1: Figure S1. Flowchart of participants**

**Additional file 1: Figure S2. Forest plots for the risk of MACE during EDIC follow-up** **by indices of intraindividual variability of body-weight during the DCCT**

Hazard Ratios (with 95% confidence interval) computed by Cox proportional hazards survival regression analysis for 1 SD of the indices of intraindividual variability of body-weight expressed as Z-scores. Forest plots on left panels (main analyses): body-weight measures from the first year following DCCT study treatment allocation not included in the computation of the indices. Forest plots on right panels (sensitivity analyses): body-weight measures from the first year following DCCT study treatment allocation included in the computation of the indices. Results shown for all participants and with stratification by DCCT study treatment allocation.

**Additional file 1: Figure S3. Forest plots for the risk of all-cause death during EDIC follow-up** **by indices of intraindividual variability of body-weight during the DCCT.**

Hazard Ratios (with 95% confidence interval) computed by Cox proportional hazards survival regression analysis for 1 SD of the indices of intraindividual variability of body-weight expressed as Z-scores. Forest plots on left panels (main analyses): body-weight measures from the first year following DCCT study treatment allocation not included in the computation of the indices. Forest plots on right panels (sensitivity analyses): body-weight measures from the first year following DCCT study treatment allocation included in the computation of the indices. Results shown for all participants and with stratification by DCCT study treatment allocation.
